# Editorial: Biocontrol of phytopathogens-recent progress for improvement in efficacy and understanding action mechanism

**DOI:** 10.3389/fmicb.2024.1407711

**Published:** 2024-07-30

**Authors:** Raja Asad Ali Khan, Syed Sartaj Alam, Md. Shahariar Jaman, Yan Li, Musharaf Ahmad

**Affiliations:** ^1^School of Breeding and Multiplication (Sanya Institute of Breeding and Multiplication), Hainan University, Haikou, China; ^2^Key Laboratory of Green Prevention and Control of Tropical Diseases and Pests, Ministry of Education (School of Tropical Agriculture and Forestry), Hainan University, Haikou, China; ^3^Department of Plant Pathology, The University of Agriculture, Peshawar, Pakistan; ^4^Department of Agroforestry and Environmental Science, Sher-e-Bangla Agricultural University, Dhaka, Bangladesh; ^5^State Key Laboratory of Vegetable Biobreeding, Institute of Vegetables and Flowers, Chinese Academy of Agricultural Sciences, Beijing, China

**Keywords:** biocontrol, phytopathogens, resistance, plant, disease

The shift away from chemical pesticides in plant disease management is increasingly influenced by public concerns over their toxicity and environmental harm, increasing restrictions on existing pesticides, and the emergence of pesticide-resistant pathogens (Rani et al., 2021). As a result, biocontrol of plant diseases has emerged as a widely recognized alternative to chemical pesticides, playing a crucial role in integrated pest approaches. This Research Topic aims to compile recent progress and achievements in the biocontrol of plant diseases and explore their action mechanisms. Among various action mechanisms, antibiotic production against plant pathogens has been reported for several biocontrol agents. For example, Maalik et al. have shown that the biocontrol bacterium *Bacillus atrophaeus* produces antimicrobial lipopeptides and, in combination with salicylic acid (SA), effectively controls blue mold disease caused by *Penicillium italicum* in lemons. The lipopeptides directly target the pathogen *Penicillium italicum*, while SA activates a defense response in the host plant, offering enhanced protection through the synergy of different mechanisms. Li et al. identified three resorcylic acid lactones, produced by the biocontrol fungus *Pochonia chlamydosporia*, effective against the plant parasitic nematode *Meloidogyne incognita*, including a new compound, monocillin-VI glycoside. Bellotti et al. revealed that *Bacillus* species can inhibit the growth and toxin production ability of *Alternaria* species. Plant disease management can also be achieved by manipulating plant rhizosphere microbes to suppress pathogenic ones, as shown by Zhang et al., who amended soil using ammonium bicarbonate to control clubroot disease in Chinese cabbage by targeting pathogenic fungus, *Plasmodiophora brassicae*.

The bacteria in the *Streptomyces* genus are known for their biocontrol effects against a variety of plant pathogens (Gowdar et al., 2018). Zhu et al. reported antimycin A1 antibiotic from *Streptomyces* bacterium, which combats the plant pathogen fungus *Rhizoctonia solani* and elaborated on its action mechanism through microscopic, physiological, biochemical, and metabolomic analysis. Moreover, Khan et al. comprehensively reviewed the action mechanisms used by *Streptomyces* bacteria, including competition for resources, antibiosis, and parasitism, while highlighting the challenges in using *Streptomyces* for plant disease management. Shen et al. identified a broad-spectrum antifungal strain of *Streptomyces graminearus* STR-1 effective against multiple pathogens, including *Magnaporthe oryzae*. They investigated its mechanisms, such as inducing plant immunity, suppressing fungal development, and producing antifungal secondary metabolites, effectively controlling rice blast disease. All these works help us better understand how we can more effectively treat phytopathogens, opening new avenues of research that will be a reference point in the near future.

## Author contributions

RK: Conceptualization, Writing – original draft. SA: Writing – review & editing. MJ: Writing – review & editing. YL: Writing – review & editing. MA: Conceptualization, Writing – review & editing.

## References

[B1] Gowdar S. B. Deepa H. Amaresh Y. S. (2018). A brief review on biocontrol potential and PGPR traits of *Streptomyces* sp. for the management of plant diseases. J. Pharmacog. Phytochem. 7, 3–7.

[B2] Rani L. Thapa K. Kanojia N. Sharma N. Singh S. Grewal A. S.. (2021). An extensive review on the consequences of chemical pesticides on human health and environment. J. Clean. Prod. 283:124657. 10.1016/j.jclepro.2020.124657

